# Sudden Cardiac Arrest in a Previously Healthy Young Male: Perimyocarditis Presenting as Myocardial Infarction With Non-obstructive Coronary Arteries

**DOI:** 10.7759/cureus.116593

**Published:** 2026-09-20

**Authors:** Marco Antonio Rodríguez Sánchez, Victor Emmanuel Gonzalez Zazueta, Luis David Beltrán Ontiveros, Jesus Miguel Figueroa Zaldivar, Ellanyn Beltrán López, José Manuel Aguilar Rubio, Estefany Adelina Angulo Lapizco

**Affiliations:** 1 Department of Cardiology, General Hospital of Culiacán, Autonomous University of Sinaloa, Culiacán, MEX; 2 Department of Clinical and Interventional Cardiology, Center for Research and Teaching in Health Sciences, Autonomous University of Sinaloa, Civil Hospital of Culiacán, Culiacán, MEX; 3 Department of Clinical and Interventional Cardiology, Hospital Angeles Culiacán, Culiacán, MEX; 4 Department of Internal Medicine, Center for Research and Teaching in Health Sciences, Autonomous University of Sinaloa, Civil Hospital of Culiacán, Culiacán, MEX; 5 Department of Internal Medicine, General Hospital of Culiacán, Autonomous University of Sinaloa, Culiacán, MEX

**Keywords:** cardiac magnetic resonance, minoca, perimyocarditis, sudden cardiac arrest, ventricular fibrillation

## Abstract

Myocardial infarction with non-obstructive coronary arteries (MINOCA) is a heterogeneous clinical entity that may mimic acute coronary syndromes despite the absence of significant epicardial coronary artery disease. Inflammatory myocardial conditions, such as myocarditis and perimyocarditis, represent important non-ischemic causes of MINOCA and may present with life-threatening complications, including malignant ventricular arrhythmias and sudden cardiac arrest.

We report a case of a 37-year-old previously healthy male who presented with chest discomfort, syncope, and sudden cardiac arrest due to ventricular fibrillation. Post-cardiac arrest electrocardiography revealed extensive anterior ST-segment elevation, with markedly elevated cardiac biomarkers and transient severe left ventricular systolic dysfunction. Coronary angiography demonstrated non-obstructive epicardial coronary arteries, leading to the diagnosis of MINOCA. Cardiac magnetic resonance imaging revealed late gadolinium enhancement in a pattern consistent with perimyocarditis. The patient required advanced life support and intensive care management for cardiogenic shock, followed by progressive clinical improvement with complete recovery of left ventricular function.

This case highlights perimyocarditis as a rare but critical cause of MINOCA presenting with sudden cardiac arrest and malignant ventricular arrhythmias in a young patient. It underscores the importance of a systematic diagnostic approach and emphasizes cardiac magnetic resonance as a key tool for etiological diagnosis, guiding appropriate management and enabling full functional recovery.

## Introduction

Myocardial infarction with non-obstructive coronary arteries (MINOCA) represents a heterogeneous clinical entity, accounting for approximately 5-10% of all patients presenting with acute myocardial infarction [1]. Despite the absence of significant epicardial coronary obstruction, MINOCA is associated with substantial morbidity and mortality, underscoring the importance of identifying its underlying etiology to guide appropriate management [2].

Among the potential causes of MINOCA, myocarditis and perimyocarditis are important non-ischemic conditions that may clinically and electrocardiographically mimic acute coronary syndromes, including ST-segment elevation myocardial infarction (STEMI) [3]. These entities are particularly relevant in younger patients without traditional cardiovascular risk factors and may be preceded by a recent viral or upper respiratory tract infection [4]. Importantly, MINOCA should be considered a working diagnosis rather than a definitive etiological diagnosis, and the identification of non-obstructive coronary arteries should prompt further evaluation to determine the underlying cause. Myocarditis and perimyocarditis may ultimately explain the clinical presentation despite an initial suspicion of acute coronary syndrome. Furthermore, myocarditis may present with severe complications, such as malignant ventricular arrhythmias, cardiogenic shock, and sudden cardiac arrest, even in the absence of structural coronary disease [5].

Current international guidelines emphasize the role of a systematic diagnostic approach in MINOCA, incorporating early coronary angiography, followed by advanced cardiac imaging [6]. Cardiac magnetic resonance (CMR) imaging has emerged as a key non-invasive modality for etiological assessment, allowing differentiation between ischemic myocardial injury and inflammatory or other non-ischemic patterns through tissue characterization techniques, including myocardial edema and late gadolinium enhancement (LGE) [7]. Ischemic myocardial injury typically produces LGE with a subendocardial or transmural distribution corresponding to a coronary vascular territory, whereas inflammatory myocardial injury generally demonstrates a non-ischemic distribution, most commonly involving the subepicardial or mid-myocardial layers and not conforming to a single coronary territory. The revised Lake Louise criteria further improve the diagnostic assessment of acute myocarditis by integrating CMR evidence of myocardial edema and non-ischemic myocardial injury [7-10].

This case highlights the unusual and life-threatening presentation of perimyocarditis in a previously healthy young adult, characterized by ventricular fibrillation and sudden cardiac arrest, extensive ST-segment elevation, cardiogenic shock, and non-obstructive coronary arteries, with subsequent CMR demonstrating inflammatory myocardial injury. The case emphasizes the importance of recognizing inflammatory myocardial disease as an alternative diagnosis in patients initially presenting with a MINOCA-like clinical picture and highlights the diagnostic value of early CMR-based tissue characterization.

## Case presentation

A 37-year-old male patient, previously healthy, with no history of chronic degenerative diseases, substance abuse, or known cardiovascular disease, was evaluated. The only relevant medical history was a recent upper respiratory tract infection approximately 15 days before admission.

Prior to arrival at the emergency department, the patient experienced dizziness, a sensation of adrenergic surge, chest discomfort, and dyspnea at rest, followed by a transient loss of consciousness with spontaneous recovery. Subsequently, he developed worsening fatigue and chest tightness, prompting transfer to the emergency department. During transport, he experienced another episode of loss of consciousness, and upon arrival, he was found to be pulseless in cardiac arrest.

High-quality cardiopulmonary resuscitation was immediately initiated. The initial rhythm was ventricular fibrillation (Figure 1), requiring two electrical defibrillations. Return of spontaneous circulation was achieved after approximately 10 minutes of resuscitation. No recurrent episodes of ventricular fibrillation or other malignant ventricular arrhythmias were documented during the subsequent hospitalization.

**Figure 1:**
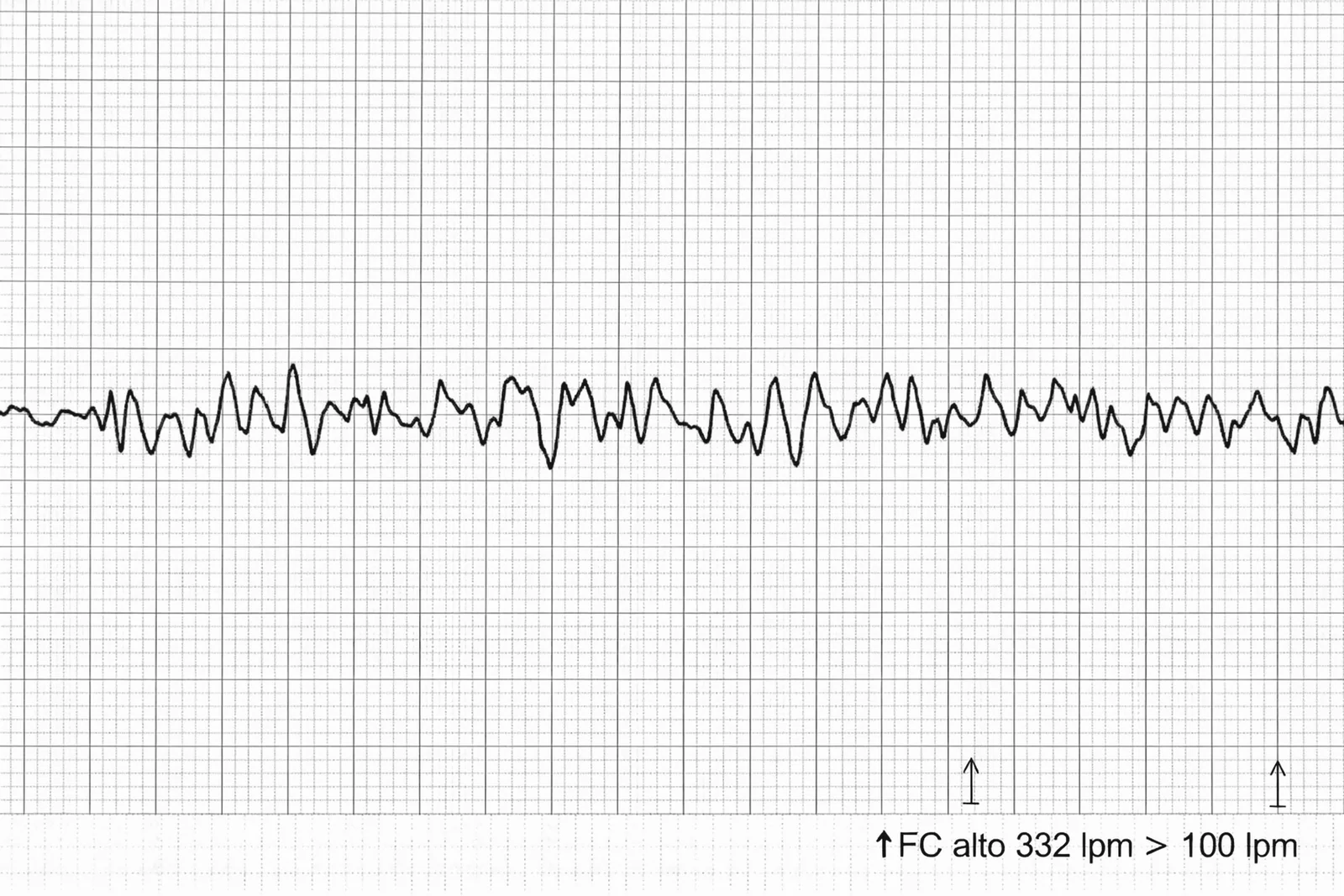
Initial electrocardiogram. Electrocardiographic documentation of ventricular fibrillation as the initial rhythm during cardiac arrest.

Immediately after return of spontaneous circulation, the patient was hypotensive, with a blood pressure of 70/30 mmHg, requiring vasopressor support. He was afebrile, mechanically ventilated following airway protection during cardiac arrest, and maintained on minimal ventilatory settings. His heart rate was approximately 90 beats/min. Focused cardiovascular examination after return of spontaneous circulation revealed no jugular venous distension, well-aerated bilateral lung fields, a regular precordium without additional murmurs, no peripheral edema, and delayed capillary refill of approximately five seconds.

Initial blood samples were obtained before the first electrical defibrillation. Initial laboratory evaluation demonstrated marked elevation of inflammatory and myocardial injury biomarkers. C-reactive protein was 190 mg/L, and erythrocyte sedimentation rate was 70 mm/h. No significant electrolyte abnormalities were identified; sodium was 138 mmol/L, potassium was 4.2 mmol/L, chloride was 100 mmol/L, phosphorus was 4.19 mg/dL, magnesium was 2.1 mg/dL, and calcium was 9.87 mg/dL. Initial high-sensitivity cardiac troponin was 6601 pg/mL, with an initial increase to 8200 pg/mL, followed by a progressive decline to 4035, 1500, 600, and 54 pg/mL. Pro-B-type natriuretic peptide was also markedly elevated initially and subsequently demonstrated a progressive decline, reaching 250 pg/mL (Table 1).

**Table 1:** Initial laboratory findings on admission. * Reference values may vary according to laboratory-specific methods and assay characteristics. The upper reference limit for high-sensitivity cardiac troponin corresponds to the 99th percentile of the assay used at our institution. CK-MB: creatine kinase-myocardial band; NT-proBNP: N-terminal pro-B-type natriuretic peptide; ESR: erythrocyte sedimentation rate.

Laboratory parameter	Initial value	Peak value	Follow-up values
High-sensitivity cardiac troponin*	6601 pg/mL	8200 pg/mL	4035 → 1500 → 600 → 54 pg/mL
CK-MB	19.6 ng/mL	—	—
NT-proBNP	5204 pg/mL	—	250 pg/mL
C-reactive protein	190 mg/L	—	—
ESR	70 mm/h	—	—
Sodium	138 mmol/L	—	—
Potassium	4.2 mmol/L	—	—
Chloride	100 mmol/L	—	—
Phosphorus	4.19 mg/dL	—	—
Magnesium	2.1 mg/dL	—	—
Calcium	9.87 mg/dL	—	—

In the immediate post-cardiac arrest period, a 12-lead electrocardiogram demonstrated left bundle branch block with extensive ST-segment abnormalities in the anterior precordial leads. Given the clinical presentation and electrocardiographic findings, the patient was evaluated for acute coronary occlusion, including assessment according to the Sgarbossa criteria [11]. The combination of the clinical presentation, electrocardiographic abnormalities, and markedly elevated cardiac biomarkers prompted urgent coronary angiography (Figure 2).

**Figure 2:**
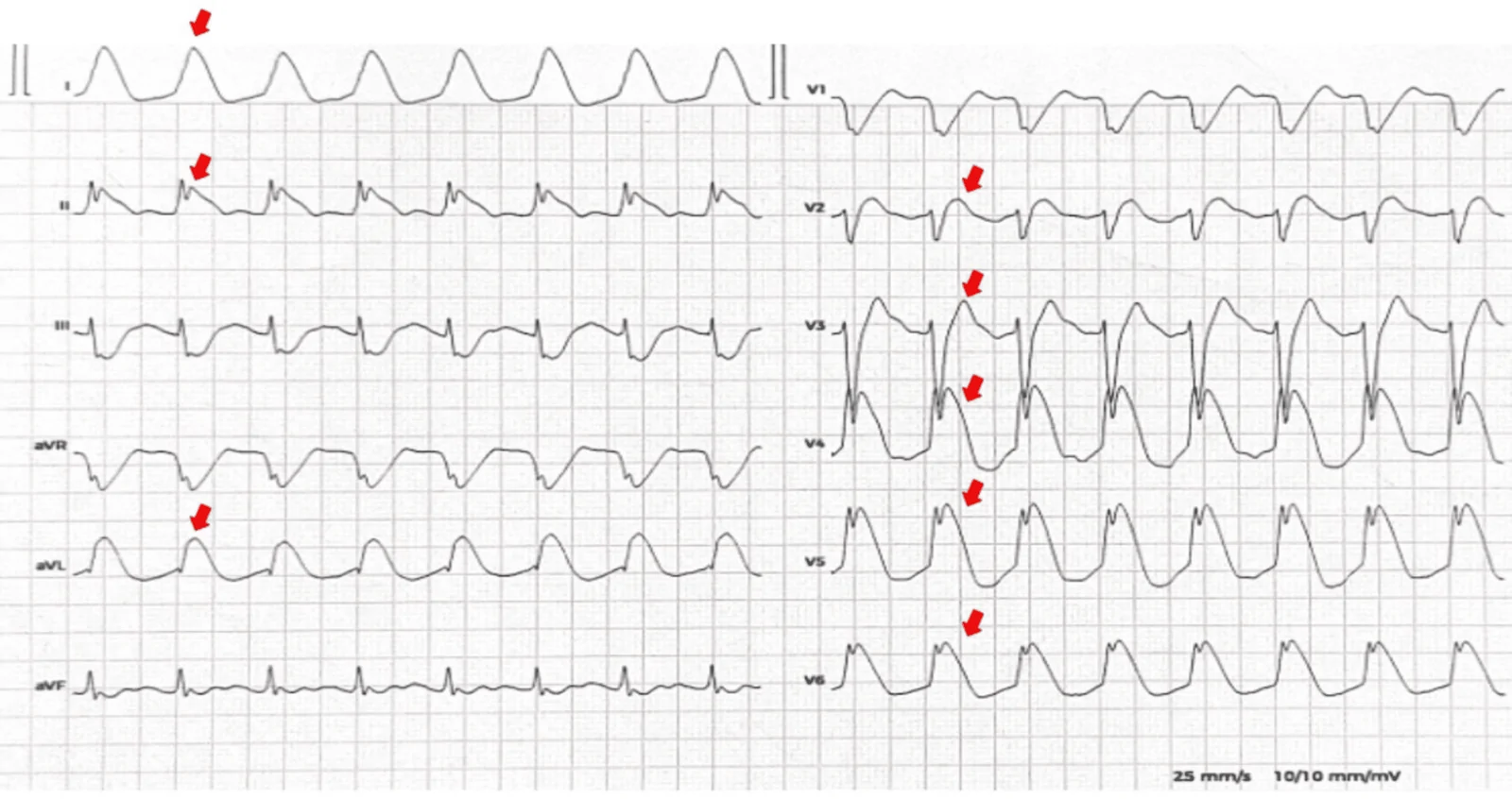
Post-resuscitation electrocardiogram demonstrating extensive anterior ST-segment elevation. Twelve-lead electrocardiogram obtained immediately after return of spontaneous circulation demonstrating left bundle branch block with extensive ST-segment abnormalities predominantly involving the anterior precordial leads (red arrows). The electrocardiographic findings were assessed according to the Sgarbossa criteria in the context of suspected acute coronary occlusion [11].

Initial transthoracic echocardiography revealed segmental wall motion abnormalities with global hypokinesia and impaired left ventricular systolic function, with an estimated left ventricular ejection fraction of 32%. Grade II diastolic dysfunction with elevated left ventricular filling pressures and mild-to-moderate mitral regurgitation were also observed (Figure 3).

**Figure 3:**
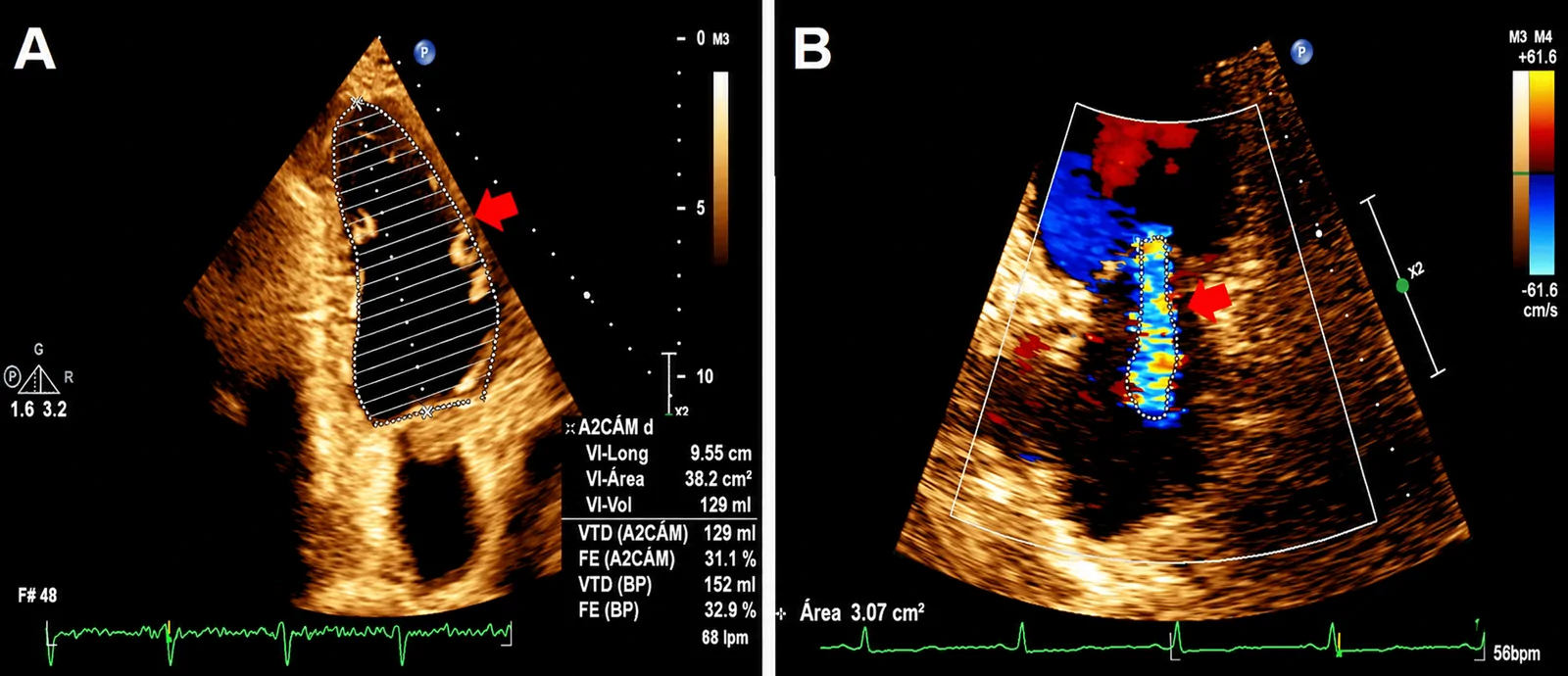
Initial transthoracic echocardiographic assessment demonstrating left ventricular systolic dysfunction. Initial transthoracic echocardiographic assessment following return of spontaneous circulation. (A) Apical two-chamber view demonstrating global hypokinesia and segmental wall motion abnormalities, with impaired left ventricular systolic function and a reduced left ventricular ejection fraction of 32% (red arrow). (B) Apical four-chamber view demonstrating mild-to-moderate mitral regurgitation (red arrow).

Given the suspicion of an acute coronary syndrome, the patient underwent coronary angiography, which demonstrated non-obstructive epicardial coronary arteries without angiographic evidence of significant stenosis (Figure 4). Based on these findings, a diagnosis of MINOCA was established, and pharmacological therapy aimed at hemodynamic stabilization of cardiogenic shock was initiated, with a favorable clinical response.

**Figure 4:**
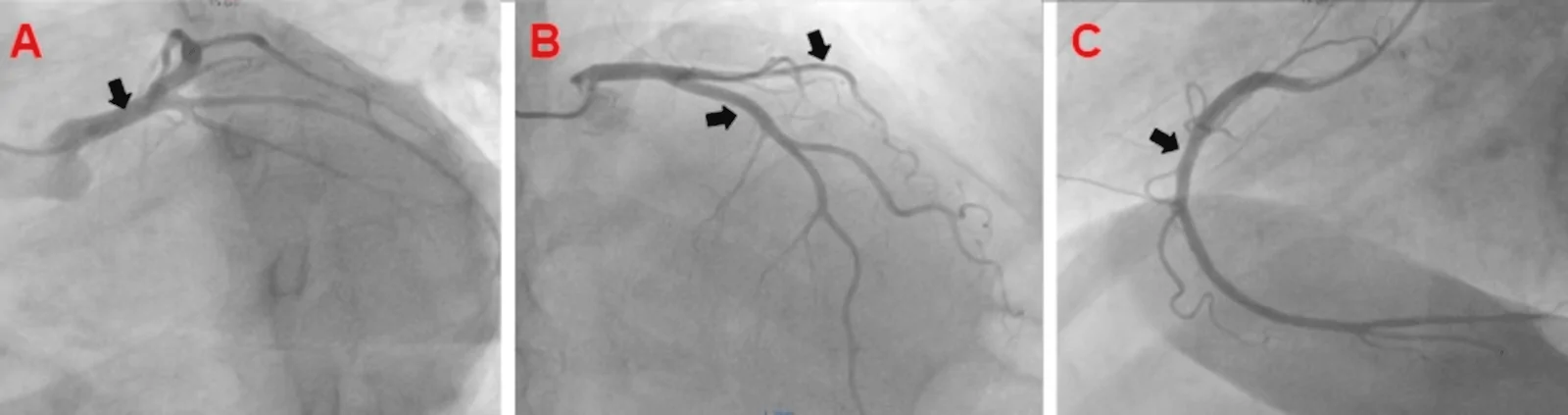
Initial coronary angiography. Coronary angiographic assessment demonstrating patent epicardial coronary arteries without significant obstructive stenosis. (A-B) Left coronary circulation showing a patent left main coronary artery, with no obstructive disease in the left anterior descending or left circumflex coronary arteries (black arrows). (C) Right coronary circulation showing a patent right coronary artery without significant obstructive disease (black arrow).

The patient was admitted to the intensive care unit for close monitoring and management of early post-cardiac arrest syndrome. Initially, he required high-dose vasopressor and inotropic support, as well as invasive mechanical ventilation with lung-protective ventilatory settings. Subsequently, he showed progressive clinical improvement, allowing for gradual tapering and discontinuation of vasopressors, cessation of inotropic support, and successful extubation 48 hours after admission.

As part of the etiological evaluation, CMR imaging demonstrated regional myocardial edema involving the basal and mid anterior, anterolateral, and lateral left ventricular segments, consistent with myocardial inflammation according to the T2-based component of the revised Lake Louise criteria [7]. LGE was present in a non-ischemic distribution, supporting myocardial injury according to the T1-based component of the revised Lake Louise criteria [7]. Thus, the combination of myocardial edema and non-ischemic LGE fulfilled the two principal CMR criteria supporting acute myocarditis. In addition, inflammatory involvement of the pericardial layers was identified, supporting the diagnosis of perimyocarditis (Figure 5).

**Figure 5:**
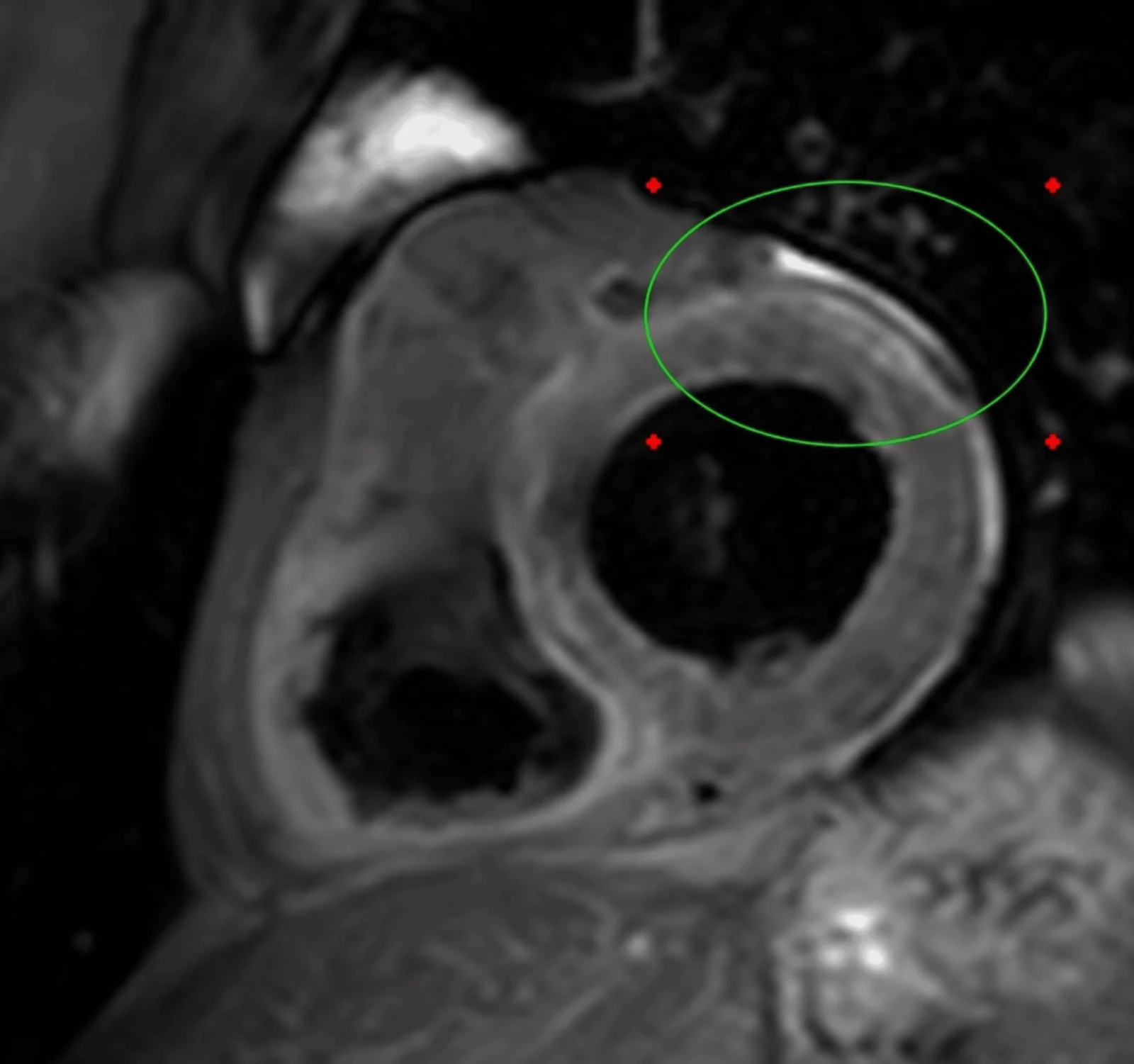
Cardiac magnetic resonance imaging. Cardiac magnetic resonance imaging demonstrating myocardial edema involving the basal and mid anterior, anterolateral, and lateral left ventricular segments (green circle), consistent with the T2-based component of the revised Lake Louise criteria. Non-ischemic late gadolinium enhancement involving the corresponding myocardial segments supports the T1-based component of the revised Lake Louise criteria [7]. Additional inflammatory involvement of the pericardial layers is also demonstrated, supporting the diagnosis of perimyocarditis.

Following the CMR findings, the initial working diagnosis of MINOCA was refined, and perimyocarditis was established as the underlying non-ischemic etiology of the patient's STEMI-like presentation.

A follow-up echocardiogram performed after extubation demonstrated significant improvement in left ventricular function, with normalization of chamber dimensions, preserved segmental wall motion at rest, and a left ventricular ejection fraction of 57%, without evidence of valvular disease (Figure 6).

**Figure 6:**
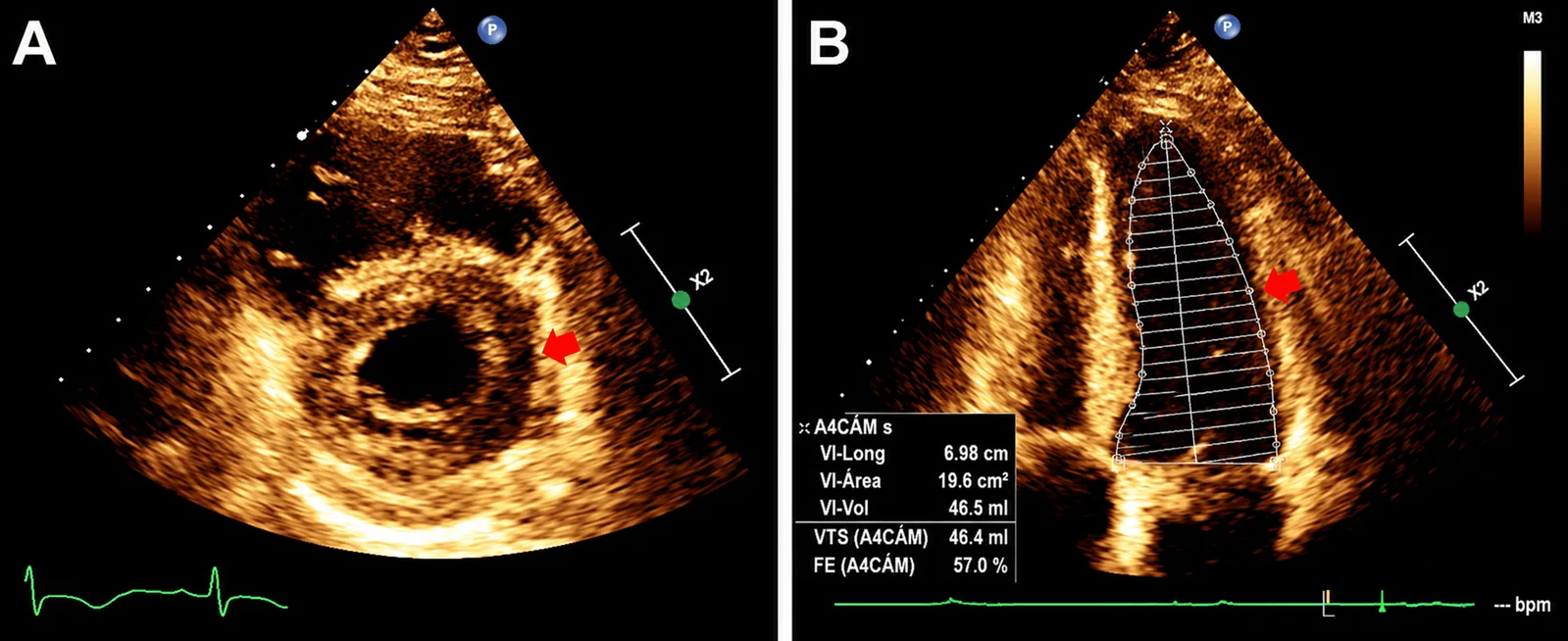
Follow-up transthoracic echocardiographic assessment demonstrating recovery of left ventricular systolic function. Follow-up transthoracic echocardiographic assessment after extubation demonstrating significant recovery of left ventricular systolic function. (A) Parasternal short-axis view demonstrating preserved and adequate segmental wall motion at rest (red arrows). (B) Assessment of left ventricular ejection fraction demonstrating recovery of systolic function, with an estimated left ventricular ejection fraction of 57% (red arrow). No significant valvular abnormalities were identified.

Likewise, a follow-up electrocardiogram showed resolution of ischemic changes, with no evidence of ongoing myocardial injury (Figure 7). The patient exhibited a satisfactory clinical course under pharmacological therapy for heart failure and myocarditis, in accordance with the recommendations of the European Society of Cardiology [12].

**Figure 7:**
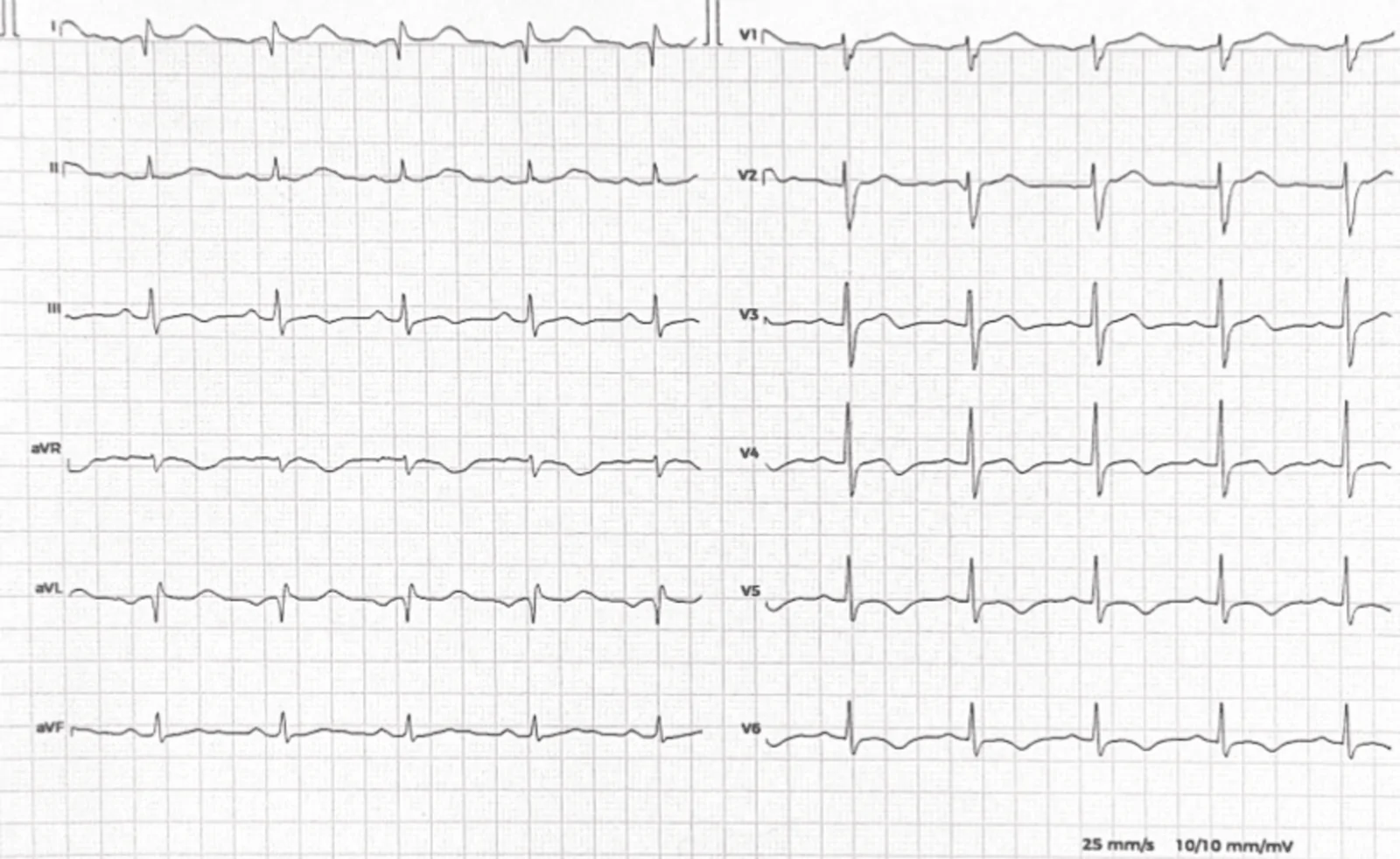
Follow-up electrocardiogram. Follow-up electrocardiogram demonstrating resolution of ischemic changes, with no evidence of ongoing myocardial injury.

Given the favorable clinical evolution, the patient was discharged home with outpatient management for heart failure, nonsteroidal anti-inflammatory drugs for the treatment of perimyocarditis, and strong emphasis on enrollment in a comprehensive cardiac rehabilitation program.

## Discussion

MINOCA constitutes a diagnostic and therapeutic challenge due to its heterogeneous etiologies and variable clinical presentations [1,2]. In the present case, a previously healthy young adult presented with ST-segment elevation, markedly elevated cardiac biomarkers, ventricular fibrillation, and cardiogenic shock, initially mimicking an acute coronary syndrome. The absence of obstructive epicardial coronary disease on angiography prompted further etiological investigation, consistent with current guideline recommendations [6].

Inflammatory myocardial diseases, such as myocarditis and perimyocarditis, are important non-ischemic causes of MINOCA [3]. These conditions typically affect young patients without cardiovascular risk factors and are often preceded by a recent viral or upper respiratory tract infection [4]. In recent studies, myocarditis has emerged as one of the most frequently identified etiologies after systematic CMR evaluation in MINOCA cohorts, reinforcing the importance of advanced imaging techniques in these patients [8].

Despite the absence of coronary artery disease, inflammatory myocardial disorders may lead to severe complications, including malignant ventricular arrhythmias, cardiogenic shock, and sudden cardiac arrest [5]. Previous studies have demonstrated that ventricular arrhythmias occurring during acute myocarditis are associated with worse short-term outcomes and may represent an important marker of disease severity requiring close monitoring and intensive care management [9].

In this patient, the presence of extensive ST-segment elevation, ventricular fibrillation, and transient severe left ventricular dysfunction suggests predominant myocardial involvement, while CMR findings supported associated pericardial inflammation, leading to the diagnosis of perimyocarditis. This overlap highlights the clinical spectrum of inflammatory cardiac disease and underscores the limitations of relying solely on clinical or electrocardiographic findings for etiological differentiation.

CMR imaging plays a central role in the diagnostic workup of MINOCA and inflammatory myocardial diseases [7]. According to current recommendations, CMR allows accurate tissue characterization through myocardial edema imaging and LGE patterns [6,7]. Additionally, the updated Lake Louise criteria have significantly improved diagnostic performance, increasing sensitivity while maintaining high specificity for detecting myocardial inflammation [10]. In the present case, CMR was pivotal in establishing the inflammatory etiology, thereby guiding appropriate management and avoiding unnecessary long-term antithrombotic or revascularization strategies.

Another notable aspect of this case is the complete recovery of left ventricular systolic function after early hemodynamic stabilization and guideline-directed medical therapy, consistent with prior evidence indicating that acute inflammatory myocardial dysfunction may be reversible when promptly recognized and adequately treated [5]. Nonetheless, the occurrence of ventricular fibrillation and cardiogenic shock highlights that inflammatory cardiac conditions are not benign and mandate close monitoring in an intensive care setting.

Overall, this case illustrates the importance of maintaining a high index of suspicion for myocarditis and perimyocarditis in young patients presenting with MINOCA, particularly when accompanied by malignant arrhythmias or hemodynamic compromise. A structured diagnostic approach, incorporating early coronary angiography, followed by CMR-based tissue characterization, is essential to ensure accurate diagnosis, guide targeted therapy, and improve clinical outcomes.

## Conclusions

Acute perimyocarditis can present with a severe acute coronary syndrome-like phenotype, including extensive ST-segment elevation, marked myocardial injury, ventricular fibrillation cardiac arrest, cardiogenic shock, and non-obstructive coronary arteries. In this setting, non-obstructive coronary angiography should prompt further etiological evaluation rather than be considered the final diagnosis. In our patient, CMR provided important diagnostic tissue characterization, demonstrating myocardial edema, a non-ischemic LGE pattern, and pericardial inflammatory involvement, supporting the diagnosis of perimyocarditis as the non-ischemic explanation for the initial MINOCA-like presentation. Recovery of left ventricular systolic function and resolution of the electrocardiographic abnormalities were subsequently observed during follow-up. This case highlights the diagnostic value of CMR in patients presenting with an acute coronary syndrome-like presentation and non-obstructive coronary arteries, particularly when myocarditis or perimyocarditis is suspected.
